# Determinants of utilization of antenatal care services in Benishangul Gumuz Region, Western Ethiopia: a study based on demographic and health survey

**DOI:** 10.1186/s12884-019-2259-x

**Published:** 2019-04-03

**Authors:** Kassahun Tiruaynet, Kindie Fentahun Muchie

**Affiliations:** 1 College of Social Science and Humanities, Debre Berhan University, Debre Berhan, Ethiopia; 2 Department of Epidemiology and Biostatistics, School of Public Health, College of Medicine and Health Sciences, Bahir Dar University, Bahir Dar, Ethiopia

**Keywords:** Utilization, Antenatal care, Determinants, Benishangul Gumuz region, Ethiopia

## Abstract

**Background:**

Utilization of antenatal care (ANC) in Ethiopia was low. There was also a variation of this underutilization of ANC service from region to region in the country. Therefore, this study aimed at providing a comprehensive analysis on socio-economic and demographic determinants of utilizing ANC services in Benishangul Gumuz Region.

**Methods:**

The study was conducted based on the 2011 Ethiopian Demographic and Health Survey. Data analyzed were taken from 674 mothers in Benishangul Gumuz Region who had at least one child in the 5 years before the survey was undertaken. Binary logistic regression model was used to analyze the data. Variables with a *p*-value< 0.25 in bivariable analysis were considered for the final model. In the final multivariable model, a variable was declared as significantly associated with ANC utilization for *p*-value< 0.05. Adjusted odds ratio with its respective 95% confidence interval was reported to show the strength of association.

**Results:**

The results of the study showed that educational level, place of residence, ethnicity, and household’s wealth were determinants of ANC utilization in the region at 5% level of significance.

**Conclusion:**

Lower educational level, difference of ethnicity, lower wealth status and rural residence of women were determinants on ANC utilization. Providing awareness creation on ANC visits for rural women during health care services provision, at community meeting, at working place and at any social association were recommended. In addition, creating conducive environment in entrepreneurial activities for poor women would improve ANC utilization. Lastly, further researches were recommended to study the effect of various traditional, cultural and other related practices on utilization of ANC services among ethnic groups in the region.

## Background

Antenatal care (ANC) is the health services provided to pregnant women by skilled health professionals before babies were born for reducing maternal mortality and morbidity rate [1–5]. The components of ANC includes: risk identifications; prevention and management of pregnancy related diseases; and health education and health promotion [6]. The World Health Organization recommends a minimum of four antenatal care visits during pregnancy [7].

Maternal mortality is one of the major development and health challenges facing the developing world. Accordingly, about 99% of global maternal mortality in each year occurred in developing countries [8]. Recently, approximately 800 women die of pregnancy related complications every day worldwide [9]. Ninety-nine percent of these deaths occur in developing countries, and Sub-Saharan Africa alone accounted about two thirds (62%) of the deaths [9]. Ethiopia is one of Sub-Saharan African countries where 19,000 maternal deaths are annually registered [10].

Despite the efforts by the government to improve maternal health using various strategies [11], Ethiopia is one of the countries with the highest maternal mortality ratios in the world. These strategies [11] have been implemented with a key intervention to bring women who were not able to reach to health facilities for maternity care services, and to encourage providers to deliver services. Though these strategies have been employed, improvement in maternal and perinatal mortality in Ethiopia is minimal over time [12]. Similarly, the Ethiopian maternal mortality ratio per 100, 000 live births were 676 in 2011 [13] and 412 in 2016 [14] which is far from targeted reduction to 199/100,000 live births [11]. However, the majority of perinatal deaths were attributed to mechanical causes of low levels of skilled attendance, improving access to obstetric care in Ethiopia [12].

Although a minimum of four ANC visits are recommended [7], only around 50% of women in developing countries received adequate ANC [15]. In Ethiopia, only about 32% of the women had four or more ANC and 64% of the women had at least one ANC visits while about 36% women did not receive any ANC, during the length of their pregnancy [13]. The above figures showed that ANC service utilization in Ethiopia is still by far below any acceptable standard. Hence, the problem still remains a challenge in Ethiopia.

A countrywide report of Ethiopian Demographic and Health Survey (EDHS) 2011 also depicted that the utilization of ANC services among regional states of Ethiopia was uneven. The EDHS data reported that 93.6, 57.2, 55.9, 54.5 and 35.1% were proportion of women who utilized ANC services in Addis Ababa, Dire Dawa, Harari, Gambella and Benishangul Gumuz respectively [13]. Utilization of ANC services in Benishangul Gumuz region was low as compared to other parts of Ethiopia mentioned above.

Previous studies covered small geographical areas, which were unable to identify associated factors of utilization of ANC at regional level, Benishangul Gumuz Region. For instance, earlier studies conducted in Metekel Zone [16] and Assosa District [17] were good examples of studies in small coverage of areas in the study region. In addition, factors affecting utilization of ANC in Benishangul Gumuz Region were not documented so far using evidence from 2011 demographic and health survey so far. Thus, this study covered a wide geographical area at Benishangul Gumuz Region, Western Ethiopia.

In order to improve utilization of ANC services in Benishangul Gumuz Region it is necessary to conduct researches to identify the determinants of utilization of ANC services in the region. Therefore, this study tried to identify socio-economic and demographic factors affecting utilization of ANC services in the region.

## Methods

### The study design and data

The study was based on the 2011 EDHS. The 2011 EDHS is a nationally representative, stratified and self-weighting probability sample of women aged 15–49 years. The survey was conducted by the Central Statistical Agency with technical assistance from ORC Macro. The detailed descriptions of methods of the survey are given in the report [13]. The EDHS 2011 covered 16,515 women aged 15–49 years. Of these women, 1259 were in Benishangul Gumuz Region. Informations regarding ANC services for all live births in the 5 years, from 2005 to 2011, preceding the survey were collected. Accordingly, 916 women in Benishangul Gumuz Region who had at least one child during last 5 years were interviewed. Out of these, 674 women had complete information for the variables considered in this study. However, the remaining women who had incomplete information were excluded as a means of treating missing values.

### Outcome variable and method of analysis

The data were analyzed by using the statistical package for social sciences (SPSS) version 20 software. The outcome variable considered in this analysis was a binary indicator: utilization of ANC from skilled health personnel, at least one of the components of ANC services. A woman was considered as utilized if she takes any of the ANC services provided by skilled health personnel. Here skilled personnel include Nurses, Midwifes, Doctors and Health Officers.

Descriptive statistics including percentages and frequencies were used to describe the study participants. Binary logistic regression model was employed to determine the determinants of utilization of ANC. Binary logistic regression model is a generalized linear model for dichotomous response variable that is, the response variable taking the value 1 with a probability of success *π*. The explanatory or predictor variables can take any form. The relationship between explanatory variables and response variable was non-linear, but rather a linear relationship exists between the explanatory variables and logit of the response variable. Given P explanatory variables denoted by vector ***X***^′^ = (*X*_1_, *X*_2_, *X*_3_,  … , *X*_*p*_) the conditional probability that the outcome occurs is denoted by *P*(*Y* = 1| *X*) = *π*(*x*). The logistic regression model is:

$$ logit\left[\pi (x)\right]=\ln \left[\frac{\pi (x)}{1-\pi (x)}\right]={\beta}_0+{\beta}_1{x}_1+{\beta}_2{x}_2+\dots +{\beta}_p{x}_p $$

Where, ${\beta}_i^{\prime }$ s for *i* = 0, 1, 2, … . , *p* are constant and coefficients of the model.

A bi-variable chi-square test of association between one explanatory variable and the outcome variable was used to select variables for the final multivariable binary logistic regression. Variables with a *p*-value< 0.25 were considered for the final model. In the final multivariable model a variable was declared as significantly associated with ANC utilization if p-value< 0.05. Adjusted odds ratio (AOR) with its respective 95% confidence interval (CI) was reported to show the strength of association.

## Results

### Characteristics of the participants

Socio-demographic and economic variables are presented in Table 1. Among all individuals considered for this study, nearly three-quarters (70.4%) of the women were aged 20–34 years (Table 1). On the other hand, high portions of women were from ethnic groups of Amhara, Berta and Gumuz with their respective percentages of 27.6%, 27.2% and 20.3%. Almost majority of women (90.7%) resided in rural areas and 72.8% had no formal education. Out of rural residents, 65.6% did not receive ANC from skilled health professional.

**Table 1:** Distribution of ANC by levels of socio-economic and demographic variables in Benishangul Gumuz Region

			Received ANC from skilled health personnel	
Variables	Categories	Counts (%)	No	Yes
			Percentage	Percentage
Age	15–19	52 (7.7)	27 (51.9)	25 (48.1)
	20–24	150 (22.3)	73 (48.7)	77 (51.3)
	25–29	200 (29.7)	124 (62)	76 (38)
	30–34	124 (18.4)	84 (67.7)	40 (32.3)
	35–39	93 (13.8)	68 (73.1)	25 (26.9)
	40–49	55 (8.2)	44 (80)	11 (20)
Religion	Others	30 (4.5)	26 (86.7)	4 (13.3)
	Muslim	373 (55.3)	230 (61.7)	143 (38.3)
	Orthodox	167 (24.8)	95 (56.9)	72 (43.1)
	Protestant	104 (15.4)	69 (66.3)	35 (33.7)
Ethnicity	Others	69 (10.2)	46 (66.7)	23 (33.3)
	Amhara	186 (27.6)	71 (38.2)	115 (61.8)
	Gumuz	137 (20.3)	114 (83.2)	23 (16.8)
	Berta	183 (27.2)	127 (69.4)	56 (30.6)
	Oromo	99 (14.7)	61 (61.6)	38 (38.4)
Educational status	Non-educated	491 (72.8)	342 (69.7)	149 (30.3)
	Educated	183 (27.2)	78 (42.6)	105 (57.4)
Residence	Rural	611 (90.7)	401 (65.6)	210 (34.4)
	Urban	63 (9.3)	19 (30.2)	44 (69.8)
Marital status	Living with partner	632 (93.8)	394 (62.3)	238 (37.7)
	Never in union	42 (6.2)	26 (61.9)	16 (38.1)
Wealth Index	Poor	345 (51.2)	250 (72.5)	95 (27.5)
	Medium	135 (20)	83 (61.5)	52 (38.5)
	Rich	194 (28.8)	87 (44.8)	107 (55.2)

### Determinants of antenatal care services

Results of bi-variable chi-square test of association indicated that the variable marital status (*p*-value = 0.955) was not associated significantly with utilization of ANC services. As a result, all others were considered for the final model. Insignificant Hosmer and Lemeshow goodness of fit test (p-value = 0.065) shows that the final binary logistic regression model was good fit. The result of the final model showed that variables such as educational status and ethnicity of mothers as well as type of place of residence, and wealth status of the household were significant factors of utilization of ANC services at 5% level of significance (Table 2).

**Table 2:** Binary logistic regression analysis of the utilization of antenatal care services in Benishangul Gumuz region

Variables	Categories	AOR	95.% C.I. for AOR		
			*P*-value	Lower	Upper
Educational level	Not educated	0.463	0.000	0.304	0.704
	Educate (RF)^a^				
Residence	Rural	0.423	0.012	0.216	0.826
	Urban (RF)^a^				
Religion	Others	0.582	0.414	0.159	2.132
	Muslim	0.804	0.496	0.429	1.507
	Orthodox	0.536	0.078	0.268	1.073
	Protestant (RF)^a^				
Ethnicity	Others	1.489	0.312	0.689	3.218
	Amhara	3.454	0.000	1.831	6.517
	Gumuz	0.472	0.031	0.239	0.933
	Berta	0.870	0.673	0.455	1.663
	Oromo (RF)^a^				
Wealth Index	Poor	0.454	0.000	0.297	0.692
	Medium	0.645	0.086	0.391	1.063
	Rich (RF)^a^				
Age	15–19	2.292	0.091	0.875	6.000
	20–24	2.159	0.061	0.965	4.828
	25–29	1.447	0.350	0.667	3.140
	30–34	1.616	0.249	0.715	3.653
	35–39	1.054	0.905	0.445	2.495
	40–49 (RF)^a^				

Note: ^a^ reference category

## Discussion

Using the EDHS data and an appropriate modeling approach, this study further assessed factors affecting women’s utilization of ANC services in Benishangul Gumuz Region. The study reported that only 37.7% (95% CI: 34.0–41.4%) of women in fertile age group in the region received ANC services at least once from skilled health personnel. This figure showed underutilization of ANC services in the region as compared to the targeted universal utilization of ANC services.

Women’s educational level was one of the strong predictor of the utilization of ANC services in the study area. The study also revealed that odds of receiving ANC services from skilled health personnel for un-educated women was less than educated women [AOR = 0.074, 95% CI = 0.009–0.628]. This finding was consistent with a previous study done in Nairobi, Kenya [1] which revealed that non-educated women were 0.033 times less likely to fully attend antenatal care than those who have secondary and above education. Other studies like in Debre Tabor, Ethiopia [2], in Nepal [18], Wosera district in Guinea [19] and Uganda [20] supported the present study. It is argued that the more the women are educated, the better they visit antenatal clinic and receive benefits from skilled health personnel while the reverse of this beneficiaries are less likely to attend antenatal clinic and/or receive from health personnel.

Another factor for utilization of ANC in the region was place of residence. The study showed that women who lived in rural areas were less likely to receive services from skilled health personnel than urban resident women [AOR = 0.459, 95% CI = 0.222–0.951]. This finding was in line with other earlier studies done elsewhere in developing countries. For instance, a study in Nigeria revealed that rural women were less likely to attend antenatal clinic than women in urban areas [21]. Likewise, studies done in Nepal [18], Butajira in Southern Ethiopia [22], and Metekel Zone, North West Ethiopia [16] indicated that rural women were less likely to receive ANC services from skilled health personal than their urban counter parts.

Ethnicity was also found out as a factor influencing utilization of maternal health care services in both developing and developed countries. According to a study done in Ghana, ethnicity was a determinant factor of utilization of ANC. In this study, Ga,Adangme women were less likely to use ANC as compared to the Asante women in 1993 and 2003 [23]. Further, a study done in UK revealed that women of Asian origin were more likely to book late for ANC than white British women [24]. These findings were consistent with the finding of present study in Benishangul Gumuz Region, Ethiopia. This study indicated that the Amhara women were 3.5 times higher to receive ANC services from skilled health personnel than the Oromo women. On the other hand, the result revealed that the Gumuz women were less likely to receive the service from skilled health personnel than those of the Oromo women. Different ethnic groups may have different cultures, values, norms and beliefs, and these may affect the beneficiaries’ behaviors and perceptions for the use of ANC services.

Wealth status was strongly and negatively associated with utilization of ANC services in the region. The study showed that poor women were less ANC attendants than those of rich women. Similar results have been reported in the previous studies in Uganda [20] and Nepal [18] that women from less income were less ANC attendants than those of women who have better income.

## Conclusion

Lower educational level, ethnicity, lower wealth status and rural residence of women were determinants on ANC utilization. In order to improve women’s utilization of ANC services in the study area, the following points has been recommended: providing awareness creation on ANC visits for rural women when the time of health care services provision, community meeting place, working place and any other social association. The concerned government body also should also create a better environment for poor women’s participation in entrepreneurial activities since there was a high relationship between economic status and utilization of ANC service. Lastly, further researches were recommended to study the effect of various traditional, cultural and other related practices on utilization of ANC services among ethnic groups in the region.

## Abbreviations

ANC: Antenatal care
AOR: Adjusted odds ratio
CI: Confidence interval
EDHS: Ethiopian Demographic and Health Survey
SPSS: Statistical package for social sciences
